# Detecting multiple spatial disease clusters: information criterion and scan statistic approach

**DOI:** 10.1186/s12942-020-00228-y

**Published:** 2020-09-02

**Authors:** Kunihiko Takahashi, Hideyasu Shimadzu

**Affiliations:** 1 Department of Biostatistics, M&D Data Science Center, Tokyo Medical and Dental University, 1-5-45, Yushima, Bunkyo-ku, Tokyo, 113-8510 Japan; 2 Department of Mathematical Sciences, Loughborough University, Loughborough, Leicestershire UK; 3 Teikyo University Graduate School of Public Health, Tokyo, Japan

**Keywords:** Scan statistic, Information criteria, Generalized linear model, Cluster detection test, Multiple clustering

## Abstract

**Background:**

Detecting the geographical tendency for the presence of a disease or incident is, particularly at an early stage, a key challenge for preventing severe consequences. Given recent rapid advancements in information technologies, it is required a comprehensive framework that enables simultaneous detection of multiple spatial clusters, whether disease cases are randomly scattered or clustered around specific epicenters on a larger scale. We develop a new methodology that detects multiple spatial disease clusters and evaluates its performance compared to existing other methods.

**Methods:**

A novel framework for spatial multiple-cluster detection is developed. The framework directly stands on the integrated bases of scan statistics and generalized linear models, adopting a new information criterion that selects the appropriate number of disease clusters. We evaluated the proposed approach using a real dataset, the hospital admission for chronic obstructive pulmonary disease (COPD) in England, and simulated data, whether the approach tends to select the correct number of clusters.

**Results:**

A case study and simulation studies conducted both confirmed that the proposed method performed better compared to conventional cluster detection procedures, in terms of higher sensitivity.

**Conclusions:**

We proposed a new statistical framework that simultaneously detects and evaluates multiple disease clusters in a large study space, with high detection power compared to conventional approaches.

## Introduction

In the middle of the 19th century, a deadly cholera outbreak affected the Soho area of London, UK. John Snow, a British physician, plotted the cases of cholera victims on a map and identified many victims within a short distance of a water pump on Broad Street. The disease map led him to a historic landmark, with the water from the pump identified as the source of cholera [1]. However, what if other cholera victims had also clustered around another pump just 200 yards away? Would this still be considered as a single cluster or preferably another cluster with a different epicenter? Although the cause of disease or incident cannot be determined only by mapping the victims, disease maps are useful in initial investigations of disease causes. Whether the cases of diseases are scattered randomly or clustered around multiple specific centers is a long-standing question in epidemiological studies [2].

To date, detecting the tendency of a clustering incident, particularly at an early stage, is still a key challenge for practitioners in preventing severe epidemics and pandemics. Given recent rapid advancements in the utility of combined health and geographical information, the challenge has become more complex and has initiated a range of methodological developments. Based on the domain with which disease clusters are dealt, the types of disease clustering are threefold: being purely temporal, purely spatial, and spatio-temporal, for each of which different test techniques are proposed [2]. In particular, spatial clusters indicate a spatial tendency for the presence of a disease or incident, the risk of which is relatively high to other surrounding regions.

There have been many statistical tests widely used [3] for identifying meaningful spatial clusters. Amongst those techniques, a class called the general test [4] searches for clusters without any preconceived assumptions on their locations. Whether the statistical significance information of each cluster is available, however, depends on the technique employed [5]. The techniques that do not determine any statistical significance are called global clustering tests, techniques developed by Moran [6], Whitemore et al. [7], Oden [8], Tango [9], Rogerson [10] and Bonetti and Pagano [11]. In contrast, the other techniques that provide the statistical significance information, on which the present study focus, are called cluster detection tests (CDTs), including those proposed by Besag and Newell [4], Turnbull et al. [12], Kulldorff and Nagarwalla [13], Kulldorff [14], Tango [15].

Within CDTs, the circular spatial scan statistic [14] has been used extensively along with SaTScan software [16]; examples include, as part of their cancer surveillance initiative, investigating the geographical variation of breast, lung, prostate, and colorectal cancer incidences in New York State [17]. A distinctive feature of the methodology is to adopt a circular scanning window varying its size for defining potential clusters. Such a fixed shape of the scanning window could perform less effective when detecting clusters that lie in non-circular shape regions, like regions alongside a river [18]. More recent developments focus on non-circular cluster forms, employing different spatial scan statistics; examples can be found in Patil [19], Assuncao et al. [20], and Tango and Takahashi [18]. The flexibly shaped scan statistic [18] implemented in FleXScan software [21] adopts the scan approach with an exhaustive search of all cluster candidates within a given radius of any area. This approach balances out the unfeasible exhaustive search by restricting it within pre-specified neighborhoods of each area [20]. Tango and Takahashi [22] also proposed a flexible spatial scan statistic implemented with a restricted likelihood ratio. Their technique requires much less computational time compared to the original statistic and effectively detects clusters of any shape when the relative risk (RR) becomes large.

Even though such extensive methodological developments have been made, there seems to have been little attention to the accurate statistical evaluation on the simultaneous detection of multiple clusters, in other words, identifying an appropriate number of cluster regions at the same time. A significant shortcoming of previous CDTs is that they cannot provide any statistical significance information for the identified multiple clusters. Such a limitation is simply because most of the methodologies focus on “single” cluster detection while investigating the extended study space within which more than one cluster is expected. Some CDTs can be adjusted for multiple cluster detection employing spatial scan statistics [14, 23–25], by iteratively running a conventional CDT single cluster detection algorithm—it leaves out sub-regions that are already identified as disease clusters in previous iterations until satisfactory results are obtained [14]. While the detection procedure is recursively performed, the cluster of the first choice is often referred to as the “primary” cluster, while the remaining clusters are referred to as “secondary” clusters; the conventional procedure is therefore often named as the secondary-cluster procedure (SCP).

The utility of CDTs becomes challenging when evaluating the number of clusters that lie within the study region. Each iteration of cluster detection in SCPs identifies only one cluster; thus, any test statistics, including associated p-values of the iteration, are only valid for evaluating that specific cluster. As a consequence, the current conventional approaches fail to provide an accurate assessment for selected multiple clusters. Therefore, a comprehensive approach is needed. A recent study suggests that a combined approach of statistical modeling and model selection can offer a potential solution by illustrating a case study that detects purely temporal clusters with a time series model in Takahashi and Shimadzu [26]. However, it is not always straightforward if the time series framework is directly applicable to a spatial context, which involves an extra dimension. It is unable to take advantage of the ordering structure in data—time series data are one-directional along with time, from the beginning to the end, but spatial data do not possess such a clear ordering structure. It is even unclear whether a similar approach can perform with a high detection power for cluster detection and, thus, extra care is required to develop a multiple-cluster detection framework in spatial contexts.

Here, we propose a unified framework that enables simultaneous detection and evaluation of multiple spatial-clusters by combining generalized linear models (GLMs) and information criterion approaches. The framework encompasses the procedure proposed for detecting purely temporal clusters in Takahashi and Shimadzu [26] as a special case. We present an illustrative example, the hospital admission for chronic obstructive pulmonary disease (COPD) in England, available from a textbook [27], for evaluating the performance of the proposed method. The results are compared with an SCP approach. The consistency property of the proposed procedure is also investigated in a simulation study.

## Methods

**Fig. 1:**
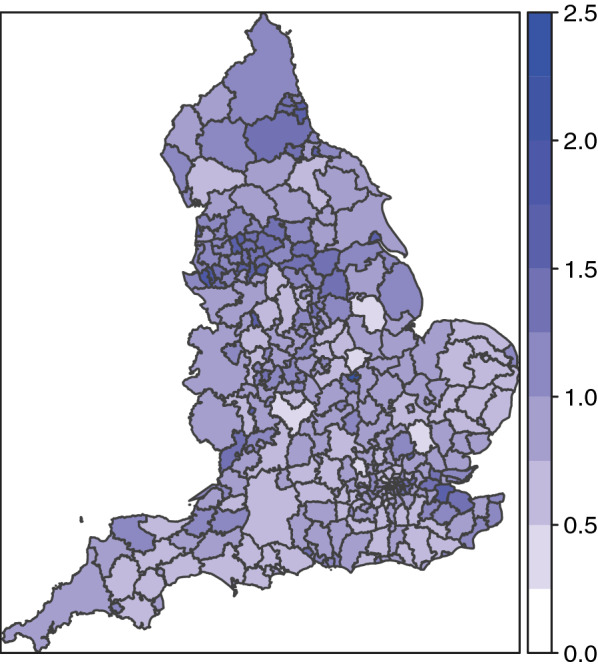
Maps of hospital admission risk of COPD in England in 2010 [27]

The proposed method will be evaluated through real and simulation data. As an illustrative example, we applied the method to the spatial distribution of the hospital admission for COPD in England for 2010 and compared the detection performance with an SCP for the spatial tendency of disease risk. COPD is a group of lung conditions that cause breathing difficulties, including emphysema and chronic bronchitis, and is common in the middle to older aged adults who smoke. Although the leading cause of COPD is smoking, some cases are due to long-term exposure to harmful fumes or dust. Figure 1 shows the spatial distribution of the risk of hospital admission for COPD. There were $m = 324$ sub-regions (local authorities) in England amongst which the total number of cases reported was 22,293. The data was taken from the book “Spatio-Temporal Methods in Environmental Epidemiology” by Shaddick and Zidek [27] (from the authors’ website: http://empslocal.ex.ac.uk/people/staff/gs454/). The color gradient corresponds to standardized admission rates adjusted by the underlying age-sex profile of the population within the sub-region; a darker color indicates a higher rate of COPD hospital admission.

A simulation study is set up to investigate the consistent property, whether the proposed method tends to select the correct number of clusters when the actual number of clusters is known. The simulation data are motivated by the COPD data to keep some reality in the spatial distribution of disease. However, the focus is given on the evaluation of detecting low RR clusters ranging from 1 to 1.6.

**Fig. 2:**
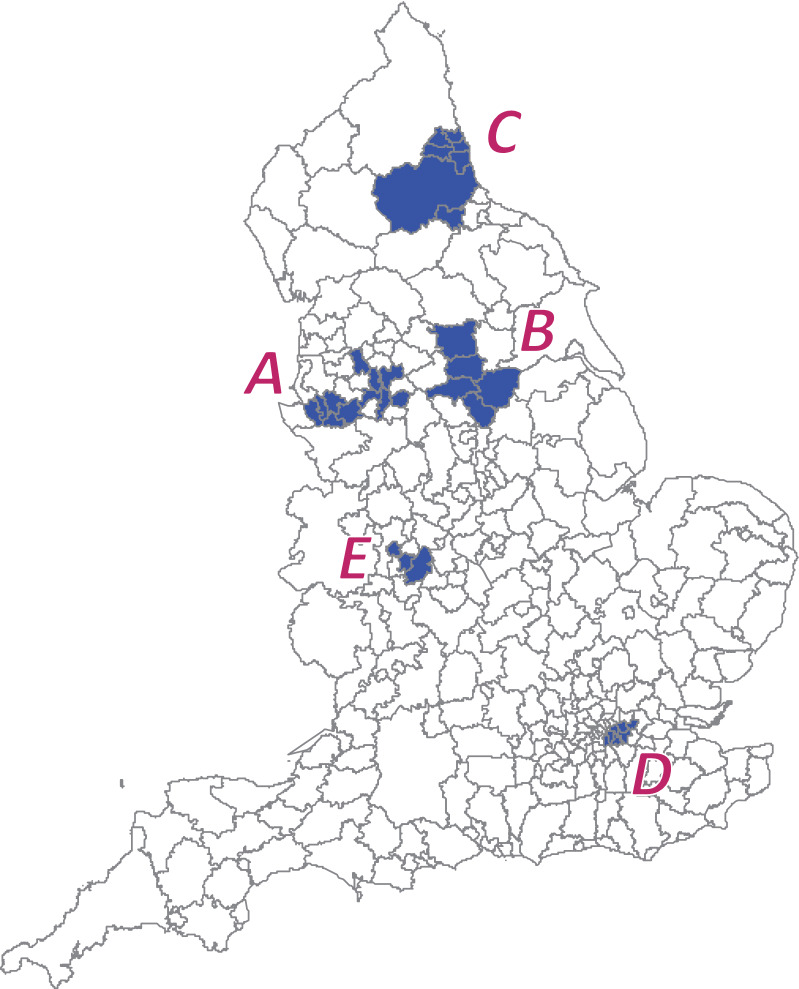
Assumed cluster areas A–E in simulation studies

In the simulation study, we assumed five clusters [A–E; Fig. 2] consisting of a different number of sub-regions, with each cluster showing a different RR according to the seven different scenarios (S1–S7) shown in Table 1. For instance, Scenario 1 (S1) indicated the null, i.e., there was no cluster, whereas Scenarios 2–5 (S2–S5) had five clusters (A–E) and Scenarios 6 and 7 (S6 and S7) assumed only single cluster (A) in the study area. For the remaining sub-regions (B–E), the RRs were set to 1.0. We generated 1000 datasets for each scenario and compared the estimated power calculated from the two cluster detection tests, the SCP and the proposed methods, at a significance level of 0.05.

**Table 1:** Assumed scenarios S1–S7 in simulation studies

	Regions	Expected Counts	Relative risk (RR)						
			S1	S2	S3	S4	S5	S6	S7
A	11	941.88	1.0	1.5	1.3	1.2	1.6	1.3	1.2
B	5	772.14	1.0	1.5	1.3	1.2	1.3	1.0	1.0
C	7	760.88	1.0	1.5	1.3	1.2	1.4	1.0	1.0
D	7	437.49	1.0	1.5	1.3	1.2	1.3	1.0	1.0
E	3	598.06	1.0	1.5	1.3	1.2	1.2	1.0	1.0
TOTAL	33	3524.31							

## Results

### Methodological developments

We first describe the challenge in detecting multiple-clusters in a spatial extent, formulating it as a mixture Poisson GLM. Here, the formulation allows that the proposed procedure directly stands on the likelihood principle and encompasses the SCP as a special case, demonstrating the critical fact that selecting appropriate multiple clusters is an exact parallel to the covariate selection in regression modeling, i.e., model selection. We then propose a new criterion for choosing a model with the appropriate number of clusters in favor of the maximum marginal likelihood, in a similar manner in deriving the Bayesian information criterion (BIC).

### Multiple-cluster model and its likelihood

Consider a study space (or area) $G$ consisting of $m$ segments (or sub-regions), each of which corresponds to the smallest element in the space (e.g., counties and states). We write the number of cases within segment $i$ as $Y_{i}$, which is assumed to follow a Poisson distribution independently with an expected value $\mu_{i}$—i.e., $Y_{i} |\mu_{i} \sim {\text{Poisson}}\left( {\mu_{i} } \right)$. And the observations (which is not random variable) of which $Y_{i}$ is denoted in lowercase as $y_{i}$, $i = 1, 2, \ldots , m$. Additionally, let ${\mathcal{W}}$ denote the set of all potential scanning zones (sets of connected segments) of any size, the construction of which set ${\mathcal{W}}$ relies on an employed scanning method. Assuming that there are $K$ clusters: $\varvec{w} = \left\{ {w_{1} , w_{2} , \ldots , w_{K} } \right\}$, in space $G$, each mutually exclusive window $w_{k}$ contains a set of adjacent segments as a cluster; i.e., $w_{k} \cap w_{{k^{\prime}}} = \phi$ for $w_{k} \ne w_{{k^{\prime}}}$. Note that $K = 0$ and $K = 1$ indicate no cluster and a single cluster in the study space, respectively.

The number of cases, $y_{i}$, is expected to be higher within hot-spot clusters compared to in other parts of the study space. The expected number of cases can be modeled as

1 $$\log \mu_{i} = \log \left( {\theta_{i} \mu_{i}^{0} } \right) = \alpha + \mathop \sum \limits_{k = 1}^{K} \beta_{k} z_{ki} + \log \mu_{i}^{0}$$

for $K \ge 1$ and $\log \mu_{i} = \alpha^{0} + \log \mu_{i}^{0}$ for $K = 0$. Here, the indicator variable $z_{ki} = 1$, if segment $i$ is a member of $k{ - }$th cluster ($i \in w_{k}$) and $z_{ki} = 0$ otherwise. Note that all coefficients are positive, $\beta_{k} > 0$. For segments that fall into the $k$-th hot-spot cluster, $w_{k}$, a parameter of model (1), becomes $\theta_{i} = \theta_{{w_{k} }} = \exp \left( {\alpha + \beta_{k} } \right)$. In contrast, for those that fall outside of the clusters ($\bar{\varvec{w}}$), the parameter is $\theta_{i} = \theta_{{\bar{\varvec{w}}}} = \exp \left( \alpha \right)$. Here, there is some flexibility in the constant term $\mu_{i}^{0} : = \mu_{i}^{0} \left( {\varvec{x}_{i} } \right)$ that is often modeled as a function of other covariates $x_{i}$, such as demographic or environmental factors; this yields the null model; i.e., the expected number of cases, when there is initially no cluster in the study space such that $\varvec{\beta}= \varvec{0}$. The null model is therefore described as $\log \mu_{i} = \alpha + \log \mu_{i}^{0}$.

The likelihood function of model (1) can be constructed as follows. Now, $f_{i} \left( {y_{i} |\varvec{z},\varvec{\psi}} \right) = f(y_{i} |\mu_{i}^{0} , \varvec{z},\varvec{\psi})$ is the probability function of $Y_{i} = y_{i}$ given the two arguments: the locations of a hot-spot window, $\varvec{z}\text{:} = \varvec{z}\left( \varvec{w} \right) = \left( {z_{ki} } \right)$, which is a $K \times m$ matrix, and the parameters $\varvec{\psi}= \left( {\alpha , \beta_{1} , \beta_{2} , \ldots , \beta_{K} } \right)$. The conditional log-likelihood function can be expressed as

$$l \left( {\varvec{\psi}|\varvec{z}} \right): = \log \left[ {\mathop \prod \limits_{i = 1}^{m} \mathop \prod \limits_{k = 0}^{K} \left\{ {f\left( {y_{i} |\mu_{i}^{0} ,\varvec{ z},\varvec{\psi}} \right)} \right\}^{{z_{ki} }} } \right],$$

  where $z_{0i} = 1$ if $i \notin \textstyle{\bigcup_{k = 1}^{K} {w_{k} }}$, and otherwise as $z_{0i} = 0$. If we assume $\varvec{z}$ to be randomly selected from a probability function $h\left( \varvec{z} \right)$, the complete (full) log-likelihood function of $\varvec{\psi}$ becomes:

$$\begin{aligned} l\left(\varvec{\psi}\right) & = \log L\left(\varvec{\psi}\right) = \log \left[ {\mathop \prod \limits_{i = 1}^{m} \mathop \prod \limits_{k = 0}^{K} \left\{ {f\left( {y_{i} , z_{ki} |\mu_{i}^{0} ,\varvec{ \psi }} \right)} \right\}^{{z_{ki} }} } \right]\\ & = l\left( {\varvec{\psi}|\varvec{z}} \right) + \log \left\{ {h\left( \varvec{z} \right)} \right\} \end{aligned}$$

where $L\left(\varvec{\psi}\right)$ is the likelihood function of $\varvec{\psi}$.

### Information criterion for selecting an appropriate $\varvec{K}$

Multiple-cluster model (1) suggests that the problem of detecting multiple clusters can be approached as a model selection problem to find an appropriate number of clusters, $K\left( { \le K_{max} } \right)$. We propose a new information criterion that chooses $K$ in favor of the maximum marginal likelihood, $ML\left( {\varvec{y},\varvec{z}} \right) = \smallint \exp \{ \log L(\varvec{\psi})\} g\left(\varvec{\psi}\right)d\varvec{\psi}$, where $g\left(\varvec{\psi}\right)$ is a prior probability function of parameter $\varvec{\psi}$. This can be achieved as follows. Applying Taylor expansion and Laplace approximations to the marginal likelihood function, it can be approximated [28] as

$$\begin{aligned} - 2\log ML\left( {\varvec{y},\varvec{z}} \right) & \approx - 2\mathop \sum \limits_{{{\text{i}} = 1}}^{m} \mathop \sum \limits_{k = 0}^{K} z_{ki} \left\{ {\log f\left( {y_{i} |\mu_{i}^{0} ,\varvec{ z},\hat{\varvec{\psi }}} \right)} \right\} - 2\log \left( {h\left( \varvec{z} \right)} \right) \\ & \quad + q\log m + \log \left| {J\left( {\hat{\varvec{\psi }}} \right)} \right| - q\log \left( {2\pi } \right) - 2\log \left( {g\left( {\hat{\varvec{\psi }}} \right)} \right) \\ \end{aligned}$$

where $\hat{\varvec{\psi }}$ is the maximum likelihood estimator of $\varvec{\psi}$,

$$J\left( {\hat{\varvec{\psi }}} \right) = - \frac{1}{m}\frac{{\partial^{2} l\left( {\varvec{\psi}|\varvec{z}} \right)}}{{\partial\varvec{\psi}\partial \varvec{\psi^{\prime}}}}{\bigg |}_{{\varvec{\psi}= \hat{\varvec{\psi }}}}$$

and $q = K + 1$. The model evaluation criterion can then be obtained by eliminating terms with an order less than $O\left( 1 \right)$ with respect to the large sample size $m$; that is,

2 $$C\left( K \right) = - 2l\left( {\hat{\varvec{\psi }} |\varvec{z}} \right) - 2\log \left( {h\left( \varvec{z} \right)} \right) + \left( {K + 1} \right)\log m, \quad \left( {K \ge 1} \right).$$

To select an appropriate number of clusters, $K$, we define a relative difference statistic based on criterion $C\left( K \right)$ as

$$RDC\left( K \right) = \left( {C_{0} - C\left( K \right)} \right)/C_{0} ,$$

where $C_{0} = C\left( 0 \right)$, the criterion under the null model. Appropriate multiple clusters are selected from the set of candidates $\tilde{\varvec{w}} = \left( {w_{1} , w_{2} , \ldots , w_{K} } \right)$ with respect to $\mathop {\hbox{max} }\limits_{K} RDC\left( K \right)$.

For the calculation of the proposed criterion (2), the probability function $h\left( \varvec{z} \right)$ must be specified. We recommend $h\left( \varvec{z} \right) = \left( {1/m} \right)^{K}$ as an approximation of the probability of selecting locations $\varvec{w}$ given the fixed windows size, shape, and direction, when the window size is relatively very small, $\# \{ i |i \in \varvec{w}\} \ll m$, with respect to the whole data size $m$. Thus, a cluster selection criterion is now given as

$$C\left( K \right) = - 2l\left( {\hat{\varvec{\psi }} |\varvec{z}} \right) + \left( {3K + 1} \right)\log m, \quad \left( {K \ge 1} \right).$$

### Statistical significance of overall clusters

The Monte Carlo hypothesis testing procedure evaluates the statistical significance of appropriate models in the same manner as the standard scan statistic. Under the null hypothesis, a large number of random datasets are generated; however, for each of these, $\mathop {\hbox{max} }\limits_{K} RDC\left( K \right)$ is instead calculated as a test statistic (see details [26]).

### Candidates of multiple clusters $\varvec{w}$

For the multiple-cluster model (1), candidate clusters, $\varvec{z}$, i.e., $\varvec{w}$ among a large number of combinations of sets in ${\mathbf{\mathcal{W}}}$, must be chosen in advance. Using an SCP method, namely the flexibly shaped scan statistic, we sequentially selected candidate clusters $w_{1}^{*} , w_{2}^{*} , \ldots , w_{{K_{max} }}^{*}$ up to the predefined maximum number $K_{max}$. While the single cluster detection procedure is iteratively applied, the cluster of the first choice, $w_{1}^{*}$, is often called the “primary” cluster, with the remaining $w_{2}^{*} , w_{3}^{*} , \ldots , w_{{K_{max} }}^{*}$ referred to as “secondary” clusters. Note that $K_{max} = 1$ corresponds to the detection of only the primary cluster. In practice, we predefine the maximum number of candidates (e.g., $K_{max} = 10, 20, \ldots$) or a $p$-value threshold, $p_{s}$ (e.g., $p_{s} < 0.5, 0.8, 1.0$) derived as the “secondary cluster” by SCPs, as there are no overlaps among the candidate clusters. The $p$-value for each cluster selected by an SCP is often calculated by the Monte Carlo hypothesis testing procedure. The selection of candidates may differ depending on the scanning method used (e.g., circular, flexible, and so forth).

### An illustrative example

**Fig. 3:**
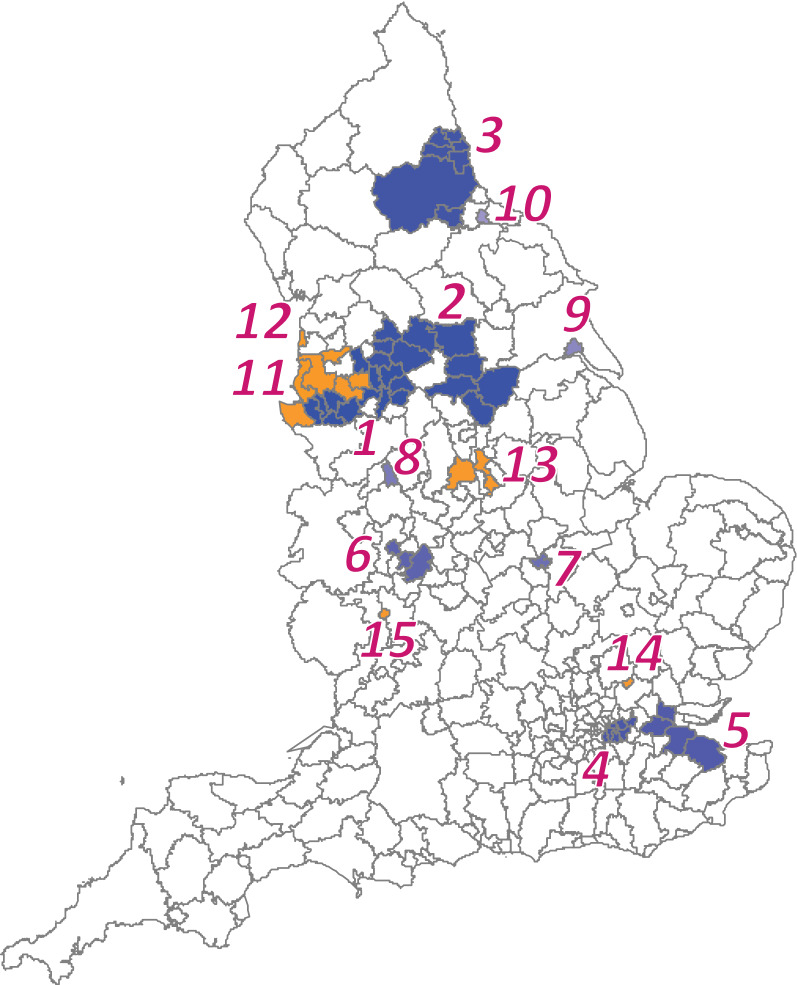
Detected clustered-areas. The blue shaded areas were detected by both the conventional SCP and proposed approaches, whereas the orange shaded areas were selected only by the proposed approach

As an illustrative example, we applied the method to the COPD data in England ($m = 324$ sub-regions) for 2010, shown in Fig. 1. A comparison of our proposed method and conventional SCP revealed a distinctive difference in the number of detected clusters. The proposed method tended to detect more clusters compared to the conventional SCP approach, as shown in Fig. 3 and Table 2. Note that some clusters are next to each other as if they are in the same single cluster, for example $w_{1}^{*} , w_{2}^{*} ,w_{11}^{*} , w_{12}^{*}$; however, they are not because their RRs differ. In the analysis, the candidate clusters $\varvec{w}$ were chosen by the restricted flexible shaped scan statistic [22] with the maximum number of the area as 20. The $p$-values were calculated by the Monte Carlo hypothesis testing procedure with 9999 replications for each cluster selected by the SCP.

**Table 2:** Detected clustered-areas with $p$-values, $p_{s}$ as the secondary, and $p_{M}$ of the multiple clusters

$w_{i}^{*}$	No. of sub-regions	Obs.	RR	Log likelihood ratio for $w_{i}^{*}$	$p_{s}$	$p_{M}$
1	11	1486	1.58	140.44	0.0001	
2	11	1598	1.31	55.95	0.0001	
3	7	1061	1.39	54.75	0.0001	
4	7	594	1.36	25.71	0.0001	
5	4	396	1.46	25.26	0.0001	
6	3	738	1.23	15.68	0.0015	
7	1	51	2.39	14.78	0.0035	
8	1	159	1.58	14.28	0.0048	
9	1	153	1.57	13.65	0.0073	
10	1	95	1.69	11.06	0.0490	
11	6	747	1.19	10.94	0.0526	
12	1	107	1.52	8.34	0.2513	
13	3	259	1.27	7.08	0.4747	
14	1	54	1.65	5.75	0.7767	
15	1	60	1.60	5.71	0.7853	0.0001

Our proposed method suggested a total of 15 clusters $\left( {w_{1}^{*} , w_{2}^{*} , \ldots , w_{15}^{*} } \right)$ with the $p$-value of the multiple cluster model as $p_{M} =$ 0.0001 ($C\left( {15} \right) =$ 2926.92 and $RDC\left( {15} \right) =$ 0.2242, where $C_{0} =$ 3724.78). In contrast, the conventional SCP detected $K =$ 10 clusters $\left( {w_{1}^{*} , w_{2}^{*} , \ldots , w_{10}^{*} } \right)$ at a significance level of $p_{s} <$ 0.05 (Table 2). Although clusters $w_{11}^{*} , w_{12}^{*} , \ldots , w_{15}^{*}$ with $p_{s} >$ 0.05 were excluded by the conventional SCP approach, the proposed method suggested that they should be included, as the $p$-value of the multiple cluster model was $p_{M} =$ 0.0001.

### Simulation study

Table 3 shows the number of detected significant multiple clusters $K$ of the SCP and proposed procedure along with the total power among 1000 datasets for each scenario. Note that the RRs of S5 were set to resemble those of the first five clusters in the example data (Table 1). Table 4 shows the sensitivity (Sen) and positive predictive value (PPV) of regions detected as significant, as well as their averages and number of detections with Sen = 1 and PPV = 1 among the 1000 datasets.

**Table 3:** The number of detected significant multiple clusters $K$ of the secondary-cluster procedure (SCP) and proposed procedures in the simulation study

$K$	1	2	3	4	5	6	7	8	Power/size ($\times$ 1000)	Number of N.S. ($K = 0$)
S1 (no cluster): RR = 1.0										
SCP	36	0	0	0	0	0	0	0	36	964
Proposed	24	8	0	0	0	0	0	0	34	966
S2 (five clusters): RR = 1.5										
SCP	0	0	0	1	997	2	0	0	1000	0
Proposed	0	0	0	0	953	45	2	0	1000	0
S3 (five clusters): RR = 1.3										
SCP	0	2	54	413	531	0	0	0	1000	0
Proposed	0	0	0	28	890	76	5	1	1000	0
S4 (five clusters): RR = 1.2										
SCP	220	385	270	97	9	0	0	0	981	0
Proposed	10	50	160	345	401	30	1	0	997	0
S5 (five clusters): RR = {1.6, 1.3, 1.4, 1.3, 1.2}										
SCP	0	29	433	495	43	0	0	0	1000	0
Proposed	0	0	20	301	621	56	2	0	1000	0
S6 (single cluster): RR = 1.3										
SCP	976	24	0	0	0	0	0	0	1000	0
Proposed	807	168	24	1	0	0	0	0	1000	0
S7 (single cluster): RR = 1.2										
SCP	914	11	0	0	0	0	0	0	925	75
Proposed	749	161	14	2	0	0	0	0	926	74

**Table 4:** Sensitivity and PPV of the secondary-cluster and the proposed procedures in the simulation study (five clusters with 33 regions)

	Detected regions (avg)	Sen (avg)	Sen = 1 (/1000)	PPV (avg)	PPV = 1 (/1000)
S2 (five clusters): RR = 1.5					
SCP	34.6	1.000	0.994	0.954	0.240
Proposed	34.8	1.000	0.996	0.950	0.232
S3 (five clusters): RR = 1.3					
SCP	33.8	0.901	0.510	0.884	0.042
Proposed	37.6	0.992	0.921	0.874	0.022
S4 (five clusters): RR = 1.2					
SCP	19.2	0.479	0.009	0.826	0.095
Proposed	33.2	0.815	0.272	0.816	0.014
S5 (five clusters): RR = {1.6, 1.3, 1.4, 1.3, 1.2}					
SCP	28.7	0.806	0.043	0.930	0.177
Proposed	35.2	0.961	0.647	0.905	0.063
S6 (single cluster): RR = 1.3					
SCP	12.0	1.000	0.999	0.930	0.446
Proposed	13.1	1.000	0.999	0.873	0.374
S7 (single cluster): RR = 1.2					
SCP	11.6	0.909	0.851	0.875	0.205
Proposed	12.7	0.912	0.855	0.822	0.173

avg: average among 1000 simulation sets; Sen: sensitivity; PPV: positive predictive value; Sen = 1: the number of detection with Sen = 1 among 1000 sets; PPV = 1: the number of detection with PPV = 1 among 1000 sets

The total powers for both procedures were very similar, except for S4. However, the SCP tended to detect a smaller number of clusters compared to the proposed method. The sensitivity of the SCP was lower than that of the proposed procedure. Notably, for weak clusters with low RRs, RR = 1.3 (S3), RR = 1.2 (S4), and mixed RRs (S5), the SCP failed to detect the five clusters with a higher power. Therefore, the sensitivity of the SCP and the probability of Sen = 1 for these scenarios were much lower than that of the proposed procedure.

In contrast, the proposed procedure tended to detect more clusters than the actual value. The PPVs of the proposed procedure were slightly lower than those of the SCP approach, but its sensitivity appeared to be higher. These simulation results suggest that the proposed procedure can detect regions within the assumed clusters with RR > 1.0 accurately with slightly extended regions. A similar performance was observed in scenarios S6 and S7 for which a single cluster was assumed.

## Discussion

Several studies have been conducted to detect multiple clusters using scan statistics other than SCPs. For example, Zhang et al. [23] proposed an adjusted $p$-value for a sequential detection approach, recursively locating clusters based upon all previously detected clusters. Although this method performs better with a higher power than conventional SCPs, the relative sizes of the adjusted $p$-values for secondary clusters are irrelevant to the order in which the clusters are sequentially detected; thus, the $k$-th cluster may have a smaller $p$-value than the previously detected $\left( {k - 1} \right)$-th cluster. Additionally, the procedure can only evaluate the significance of individual clusters but not of multiple clusters as a whole.

In the spatial context, a multiple cluster detection procedure using spatial scan statistics was described in [24, 25]. However, this method cannot assess the significance of multiple clusters as a whole. A generalized linear mixed model with Moran’s $I$ statistic and stepwise procedure allows for multiple cluster evaluation, accounting for random spatial effects. The power of the approach is lower than that of the standard scan statistic [29]. A recent study [30] suggested a quasi-likelihood approach that deals with spatial correlation. However, quasi-likelihood suffers from the multiple testing problem in selecting multiple clusters, as the approach does not provide a full-likelihood. Our approach avoids this issue by utilizing the model selection framework with the proposed information criterion based on the full-likelihood principle.

We proposed an information criterion for selecting an appropriate number of clusters. The information criterion approach is based on the framework proposed by Takahashi and Shimadzu [26] for detecting multiple temporal-clusters. The idea of model selection has been used in more general statistical modeling contexts; for instance, Akaike information criterion (AIC) and Bayesian information criterion (BIC) are used to estimate the number of multiple clusters [31, 32] and finite mixtures [33]. However, in situations where large datasets are used, conventional information criteria, including $- 2$ log likelihood, AIC, and BIC, perform poorly and cannot accurately select an appropriate number of clusters. The proposed criterion is derived from the marginal likelihood of the multiple cluster model and accounts for the probability distribution of selected candidate clusters. Our examples and simulations clearly demonstrate that the proposed criteria perform well for identifying appropriate multiple clusters.

**Fig. 4:**
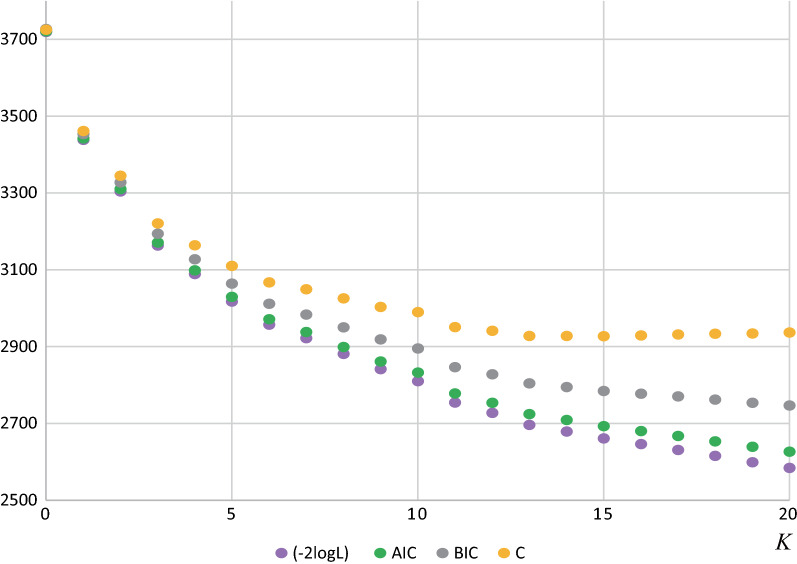
Trajectories of different model selection criteria; $- 2$ log-likelihood ($- 2$ logL), Akaike information criterion (AIC), Bayesian information criterion (BIC), and the proposed criterion $C$

Figure 4 shows the comparison of the proposed criterion $C$ with other conventional criteria: $- 2\log L$, AIC, and BIC, at $K$ ($K = 0, 1, \ldots , 20$). Although some inflection points were observed at around $K =$ 11, the proposed criterion $C$ attained a minimum value, i.e., the maximum value of $RDC$, at $K =$ 15. In contrast, other criteria monotonically decrease and do not reach minimum values for $K \le 20$.

A more conservative $p$-value is calculated by the secondary procedure as compared to the primary cluster procedure [23, 34]. Thus, the former identifies fewer significant secondary clusters relative to true clusters. This was observed in our simulation study, while the proposed procedure tends to detect more clusters, contrasting the reported result in the purely temporal setting [26], although this may largely depend on the scenario assumed.

Our case study and simulation studies demonstrate that the proposed framework performs well, although some limitations remain. First, multiple cluster detection depends on the scanning method initially used, and we adopted the conventional secondary procedure to pre-select candidate clusters for a GLM. This implies that choosing the optimal scan statistic with high detection accuracy is essential. It requires further investigations on various detection test statistics as well as other scanning methods, including the union cluster situation. Second, the spatial dependence structure must be considered for better cluster detection. These methods will provide insight for future research.

## Conclusion

We proposed a new statistical framework that combines the scan statistic and GLMs to simultaneously detect and evaluate multiple disease clusters in a large study space. The framework can determine whether the presence of a specific disease or incident is entirely random over geographical space. We also developed a new information criterion to select the appropriate number of clusters in the spatial context. Together with these approaches, the proposed framework enables the estimation and evaluation of multiple clusters with high detection power, as demonstrated in our simulation study. Further, a distinctive feature of our simultaneous detection framework is that it can calculate the $p$-value of detected multiple-clusters as a whole, as opposed to one at a time, as in conventional SCPs.

## Data Availability

The data for the risk of hospital admission for chronic obstructive pulmonary disease in the UK was taken from the book “Spatio-Temporal Methods in Environmental Epidemiology” by Shaddick and Zidek [27] (from the authors’ website: http://empslocal.ex.ac.uk/people/staff/gs454/)
